# Treatment tactic of canine cranial cruciate ligament rupture management: A 28-day comparative analysis of ACP and NSAID induced effects on the serum MMP-3 levels and clinical outcomes

**DOI:** 10.17221/39/2024-VETMED

**Published:** 2025-04-28

**Authors:** Kristina Raulinaite, Rasa Zelvyte, Kristina Skemiene, Ingrida Monkeviciene

**Affiliations:** ^1^Department of Anatomy and Physiology, Faculty of Veterinary, Veterinary Academy, Lithuanian University of Health Sciences, Kaunas, Lithuania; ^2^Laboratory of Biochemistry, Neuroscience Institute, Lithuanian University of Health Sciences, Kaunas, Lithuania

**Keywords:** autologous conditioned plasma, dog, cranial cruciate ligament rupture, functional outcomes, matrix metalloproteinase 3, nonsteroidal anti-inflammatory drugs

## Abstract

Cranial cruciate ligament rupture (CrCLR) is a common stifle joint pathology among dogs, leading to osteoarthritis and painfulness. Non-surgical treatment options often represent the usage of non-steroidal anti-inflammatory drugs for 14 days (NSAIDs), but autologous conditioned plasma (ACP) shows promising results in managing various orthopaedic conditions, decreasing inflammation, and improving the clinical outcome in dogs. This study aimed to determine the differences in MMP-3 serum levels and the clinical outcomes between differently treated cranial cruciate rupture cases. For this purpose, we used two different treatment methods for managing canine cranial cruciate ligament rupture (minimally invasive ACP injection or oral NSAIDs), and evaluated the clinical outcomes, indicating the quality of life, and the MMP-3 serum levels over a period of 28 days. The findings of this investigation indicate that ACP has better efficacy than two weeks of NSAIDs in inflammation reduction, clinical outcome improvement, and the allowance of a longer duration of activity after 28 days.

A rupture of the cranial cruciate ligament (CrCLR) is a frequent pathology affecting canine stifle joint (Johnson et al. 1994; Comerford et al. 2005; Clements et al. 2008; Taylor-Brown et al. 2015; Sellon and Marcellin-Little 2022). The pathology of CrCLR can lead to osteoarthritic changes of various degrees in the stifle (Vasseur and Berry 1992; Innes et al. 2004; Bleedorn et al. 2011; Alvarez et al. 2022). The ruptured ligament also leads to stifle instability, joint effusion, inflammation, and painfulness (Brandt et al. 1991; Rayward et al. 2004). The severity of the osteoarthritis (OA) affects the CrCLR prognosis and the method of its management (Chauvet et al. 1996; Innes et al. 2000).

The pathways affecting disturbance in the degradation and repair of the balance of cartilage matrix in OA are mediated through cytokines and chemokines (Fernandes et al. 2002). The cytokine activities correlate with the functional alterations in the synovial membrane, cartilage and subchondral bone, and are produced spontaneously or subsequently stimulated by cells in the joint tissue (Martel-Pelletier et al. 1999). Matrix metalloproteinease-3 (MMP-3) is associated with OA and is one of the most sensitive matrix metalloproteineases (MMPs) in cases of canine CrCLR (Hegemann et al. 2003; Hayashi et al. 2004; Muir et al. 2005). Biomarker MMP-3 is locally produced and activated within the affected joint as a result of the cytokine mediated stimulation. It is a sensitive biomarker of both canine and human OA (Qi and Changlin 2007).

Biomarkers are useful in evaluating OA, as they can be used to assess the proteins or enzymes directly or indirectly affecting the joint inflammation and painfulness. The serum is one of the easiest biological fluids to collect and is a suitable source of biomarkers (De Gruttola et al. 2001).

The non-surgical management of CrCLR is set to maintain the patient’s comfort levels and affected limb’s functionality while non-steroidal anti-inflammatory drugs (NSAIDs) are a part of the most common non-surgical treatment options in cases of canine CrCLR (Kealy et al. 2002; Mlacnik et al. 2006). The anti-inflammatory effect is specific for NSAIDs because they act by inhibiting the cyclooxygenase (COX) enzyme (KuKanich et al. 2012). Cimicoxib (CX) is a NSAID characterised by its high effectivity as a COX-2 inhibitor (Jeunesse et al. 2013; Kim et al. 2014).

Glucosamine and chondroitin sulphate are used to manage OA-induced painfulness (Peal et al. 2007; Steagall et al. 2007; Gupta et al. 2012; Vassalotti et al. 2017).

Autologous conditioned plasma (ACP) is a biological product consisting of an autogenous fluid containing a supraphysiological concentration of platelets (Harrison and Subcommittee on Platelet Physiology 2018; Collins et al. 2021). The platelets contain numerous growth factors that play an important role in the proliferation and differentiation of stem cells, and also in inducing healing processes (Pavlovic et al. 2016; Qian et al. 2017). Clinical studies in both human and veterinary medicine, have shown promising results that ACP might be beneficial in the treatment of various ligament pathologies of traumatic and degenerative origin (Xie et al. 2013; Kia et al. 2018; Kaux et al. 2020).

To the best of the authors’ knowledge, there are no reports evaluating the differences in the MMP-3 serum concentrations and the clinical outcomes of CrCLR in dogs managed with ACP injections compared to NSAIDs. The primary aim of this research was to evaluate the impact of two management protocols (a single intra-articular injection of ACP and 14 days of oral NSAID administration) for CrCLR on the serum MMP-3 levels and the clinical outcomes in cases of unilateral CrCLR. Based on the available published evidence, our hypothesis was that the serum MMP-3 levels would decrease in the cases of the CrCLR treatment with both methods, even after 28 days. However, the clinical outcomes after 28 days from the start of the treatment would be more favourable for the patients treated with the intra-articular ACP injection.

## MATERIAL AND METHODS

This study is the part of investigation carried on in the Dr. L. Kriaučeliūnas Small Animal Clinic and Biochemistry laboratory of the Neuroscience Institute of the Lithuanian University of Health Sciences (LSMU) between September 2019 and December 2021 (Raulinaite et al. 2023).

The procedures complied with the criteria given by the Lithuanian animal welfare regulations (No. B1-866, 2012; No. XI-2271, 2012) and the decree of the director of the State Food and Veterinary Service, the Republic of Lithuania [No. B6-(1.9)-2103, 2020]. The owners of the animals provided consent for the patients’ participation. The research was conducted in compliance with the EU legislation.

### Animals

All the dogs matched the inclusion criteria, which included a 60-day period without the administration of NSAIDs and glucosamine/chondroitin sulphate supplements prior to admission. Additionally, physical, orthopaedic, and diagnostic imaging evaluations were conducted to confirm CrCLR and to rule out the presence of any additional pathologies; each patient’s body conditioning score (BCS) was evaluated at d0 of the research (Laflamme 1997). All the owners of the enrolled dogs voluntarily opted for the non-surgical treatment option and were not blinded to the treatment groups; all the animals were blindly assigned to the treatment groups. All the dogs were diagnosed with CrCLR based on the results of the clinical, radiological, and ultrasound examinations. The dogs were divided into two groups for the study. The two groups of dogs were treated for CrCLR with either ACP (CrCLR-1, *n* = 6) or NSAIDs and supplements (CrCLR-2, *n* = 6).

CrCLR-1 group: The group consisted of six mixed-breed dogs, four females, and two males, with a mean age of 3.2 ± 1.2 years, a mean weight of 18.2 ± 5.13 kg, a mean BCS of 4.2 ± 0.75 points, and a mean period of lameness before the first visit of 3.67 ± 2.2 days.

CrCLR-2 group: The group consisted of six mixed-breed dogs, three females and three males, a mean age of 3.35 ± 1 years, a mean weight of 17.82 ± 4.57 kg, a mean BCS of 4 ± 0.89 points, and a mean period of lameness before the first visit of 3.17 ± 1.72 days. Each dog of this group was administered cimicoxib orally once per day for 14 days, with a daily dosage of 2 mg/kg; additionally, these animals were given supplements (7.2 μg of cyanocobalamin, 53.1 μg of folic acid, 2.6 mg of thiamine, 0.51 mg of riboflavin, 1.68 mg of pyridoxine, 2.26 mg of niacin, 0.71 mg of vitamin E, 55.5 mg of calcium, 44.6 mg of phosphorus, 52.5 mg of omega nutrients, 225 mg of glucosamine sulphate, 162 mg of chondroitin sulphate, and 59.94 mg of methylsulphonylmethane) orally every twenty-four hours for fourteen days, according to the recommendations of the manufacturer (Bob Martin, UK) in the form of tablets.

Clinical and orthopaedic examinations and a blood analysis for the MMP-3 levels were performed in each group three times: on the day before the start of treatment (d0), fourteen days after (d14), and twenty-eight days after the start of treatment (d28). At the start of the study, the owners were informed about the activity management guidelines they were required to adhere to at home. These guidelines prohibited the animals from engaging in any intense activities and required only supervised walks with a leash.

All the examinations in this research were performed by an orthopaedic specialist that was not blinded to both treated groups.

### Methods

#### MANUAL EXAMINATIONS

All the animals (*n* = 12) underwent clinical and orthopaedic assessments three times: at d0, d14, and d28. A clinical examination was conducted to evaluate the overall animal’s health condition and was performed according to Bragg et al. (2015). For the orthopaedic examination, the degree of lameness (0–5), the presence of stifle effusion, the cranial tibial thrust test, and the painfulness of joint manipulations were assessed. All the animals showed positive tibial thrust, indicating CrCLR. The painfulness of joint manipulations was graded on a scale of 0 to 3, with 0 indicating no pain and 3 indicating severe pain. All the evaluations were conducted and assessed by the same veterinarian who was not blinded to the treatment cohorts.

#### RADIOLOGICAL EXAMINATION

The animals underwent general anaesthesia for the radiological assessment (Raulinaite et al. 2023). The radiological evaluation was conducted one time at d0 and was not performed continuously during the study period, where standard medio-lateral and cranio-caudal projections of the stifle joint were performed. The radiological examination was conducted with the primary goal of confirming the diagnosis of CrCLR and ruling out any concurrent pathologies, such as patellar luxation, fractures, and anomalies. Via the radiological examination, stifle joint effusion was confirmed or ruled out, by evaluating its presence in the radiographs. Following the system of stifle OA evaluation, 32 variables were evaluated for each stifle, and then an overall OA score (1–4) was calculated, where 1 refers to as no OA, and 4 refers to as severe OA (Lazar et al. 2005). Changes in the bone density and integrity, patella axis, and anatomical normality of the stifle joint and its’ elements were evaluated. No animals were diagnosed with patellar luxation, bone fractures or stifle anomalies.

#### ULTRASOUND

An ultrasound examination was conducted on the affected stifle joint at three time points: d0, d14, and d28. The primary objective of the ultrasound examination was to evaluate the meniscus damage. The methodology employed was in accordance with the approach described by Mahn et al. (2005).

#### FOLLOW-UP

A follow-up consisted of completing a modified force plate validated questionnaire (Hudson et al. 2004). The questionnaire was completed during the follow-up visits. Veterinarians interviewed the dog’s owners and recorded their answers three times: at d0, d14, and d28. The owners were questioned regarding their dog’s function before and after the ACP injection/NSAIDs administration. A scale of 1 to 6 was selected (1 = worse, 6 = best) and used in this study to respond to each question of the questionnaire (Kishi and Hulse 2016). The questionnaire is presented in Table 1.

**Table 1:** The follow-up questionnaire for evaluating the patient’s mobility and quality of life

#	Question
1.	What is the willingness to play voluntarily?
2.	What is the overall activity during the day?
3.	What is the stiffness at the start of the day?
4.	What is the stiffness at the end of the day?
5.	What is an indication of lameness while walking?
6.	What is the difficulty in sitting down?
7.	What is the difficulty of laying down?
8.	What is the difficulty in rising up?
9.	What are the changes in the amount of engaging in other activities?

#### BLOOD TESTING

The whole blood testing and blood biochemistry testing (for concentrations of glucose, creatinine, urea, total protein, albumin, globulin, alanine aminotransferase, and alkaline phosphatase) were performed for all the animals at d0 as a screening method to exclude unhealthy dogs.

#### AUTOLOGOUS CONDITIONED PLASMA

Each dog in the CrCLR-1 group underwent general anaesthesia for the purpose of the preparation and administration of the ACP (Raulinaite et al. 2023). The blood samples used for the ACP were prepared and administered by orthopaedic specialist using an ACP double syringe system (Arthrex, Inc., Naples, FL). The dose of ACP was 2 ml and was aseptically injected into the affected stifle joint one time at d0.

#### ELISA

Peripheral blood samples (4 ml) were collected and prepared three times: at d0, d14, and d28 (Raulinaite et al. 2023). The concentrations of MMP-3 in the dog serum samples were measured using an antibody-based, sandwich enzyme-linked, immunosorbent assay (ELISA) kit (Abbexa^®^, USA) strictly following the manufacturer’s recommended procedures. Absorbance readings were performed at 450 nm. All the data were expressed as mean ± standard deviation (SD).

#### STATISTICAL ANALYSIS

A statistical analysis was performed using the IBM SPSS Statistics^®^ software program. The chi-square test, Kruskal-Wallis test, and the Mann-Whitney *U* test were used to evaluate the data. The level of significance was set at *P* < 0.05.

## RESULTS

### Groups

The statistical analysis showed that the CrCLR-1 and CrCLR-2 groups were homogenous. The results showed that the age, weight, BCS, and duration of lameness before the first visit did not differ between the groups (*P* > 0.05).

### Radiological examination

Stifle effusion was diagnosed in all the animals of the CrCLR-1 (*n* = 6) and CrCLR-2 (*n* = 6) groups.

The degree of OA in the CrCLR-1 group was 2.3 ± 0.82, and the OA degree in the CrCLR-2 group was 2.17 ± 0.75. The statistical analysis revealed no statistically significant correlation between the OA and the MMP-3 levels, degree of lameness, painfulness of manipulations, and follow-up scores in either the CrCLR-1 or CrCLR-2 group.

### An ultrasound examination

The ultrasonographic evaluation revealed that 33.3% (*n* = 2) of the dogs in the CrCLR-1 group had a damaged medial meniscus at d0, while 16.61% (*n* = 1) of the dogs in the CrCLR-2 group exhibited the same pathology. These findings remained consistent at d0, d14, and d28.

### MMP-3 concentration

The statistical analysis revealed a significant difference between the CrCLR-1 and CrCLR-2 groups at d28 (*P* < 0.05). The results indicated that the MMP-3 levels of the patients treated with ACP were more than two times lower at d28 when compared to d0. The MMP-3 levels did not differ between the groups at d0 and d14 (*P* > 0.05) (Table 2).

**Table 2:** The results of the MMP-3 levels of the differently treated CrCLR groups

Parameter	Group	d0	d14	d28
MMP-3 concentration	CrCLR-1	52.43 ± 36.44	33.11 ± 18.1	11.62 ± 5.81^a^
(ng/ml)	CrCLR-2	49.38 ± 31.9	31.1 ± 19.65	25.21 ± 13.74^a^

^a^Changes in the MMP-3 concentrations between the CrCLR groups at d28 (*P*** < **0.05)

d = day of treatment; CrCLR = cranial cruciate ligament rupture; MMP-3 = matrix metalloproteinease-3

### Degree of lameness

A significant difference was revealed between the degree of lameness of the CrCLR-1 and CrCLR-2 groups at d14 (*P* < 0.05), and at d28 (*P* = 0.025). At d14, the degree of lameness was reduced in both groups compared to d0, but, at d28, the patients treated with ACP continued to display a very low level or no degree of lameness, while patients treated with NSAIDs had recurrent lameness (Table 3).

**Table 3:** The results of the degree of lameness of the differently treated CrCLR groups

Parameter	Group	d0	d14	d28
Degree of lameness	CrCLR-1	4.66 ± 0.52	1.83 ± 0.75^a^	0.17 ± 0.41^b^
(0–5)	CrCLR-2	4.17 ± 0.75	0.33 ± 0.52^a^	2.0 ± 0.89^b^

^a^Changes in the degree of lameness between the CrCLR groups at d14 (*P*** < **0.05); ^b^Changes in the degree of lameness between the CrCLR groups at d28 (*P*** < **0.05)

d = day of treatment; CrCLR = cranial cruciate ligament rupture

The statistical analysis revealed no statistically significant correlation between the degree of lameness and the MMP-3 serum levels.

### Painfulness of manipulations

A statistical comparison between the painfulness of the manipulation scores of the CrCLR-1 and CrCLR-2 groups revealed a highly significant difference at d28 between the groups (*P* < 0.01). At this time point, patients treated with ACP showed almost ten times lower scores of painfulness during the orthopaedic manipulations (Table 4).

**Table 4:** The results of the painfulness of the manipulations of the differently treated CrCLR groups

Parameter	Group	d0	d14	d28
Painfulness of mani-	CrCLR-1	2.67 ± 0.52	0.5 ± 0.55	0.17 ± 0.41^a^
pulations (1–3)	CrCLR-2	3.0 ± 0.00^a^	0.67 ± 0.82	1.67 ± 0.52^a^

^a^Changes in the painfulness of the manipulations between the CrCLR groups at d28 (*P*** < **0.01)

d = day of treatment; CrCLR = cranial cruciate ligament rupture

### Follow-up

**Figure 1:**
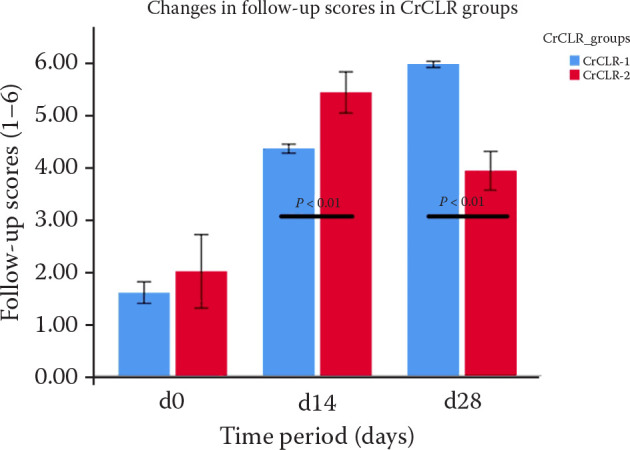
The impact of the different treatments on the follow-up scores (the mean values) at three time points (d0, d14, and d28)

All the animal owners responded to all the questionnaire items, indicating their ratings on a 1–6 scale, with 1 representing the worst possible result and 6 representing the best possible result. The statistical analysis revealed that, at d14, the mean follow-up score of the quality of life of the CrCLR-1 group was 16.6 % lower than that of the CrCLR-2 group; these differences were statistically significant (*P*** < **0.01). At d28, the mean follow-up score of the quality of life of the CrCLR-1 group became higher (5.96 ± 0.06), while the mean follow-up score of the CrCLR-2 group decreased (4.24 ± 0.35); these differences at d28 were statistically significant (*P*** < **0.01). A statistical evaluation of the data, presented as the mean of the entire questionnaire at d0, d14, and d28, revealed a continuous increase in the follow-up score within a group of the CrCLR cases treated with ACP (Figure 1).

## DISCUSSION

One of the most prevalent causes of stifle instability and OA in dogs is CrCLR (Vasseur and Berry 1992). Amongst new treatment modalities, ACP contains various growth factors and is a promising healing modality for damaged cartilages and ligaments both in the human and veterinary medicine fields (Billinghurst et al. 1997; Arnoczky et al. 2011; Canapp et al. 2016; Moussa et al. 2017; Kaux et al. 2020; Vander Doelen and Jelley 2020). The biomarker MMP-3 plays an important role in processes affecting connective tissue matrix during growth. Abnormal MMP-3 production causes cartilage matrix degeneration during OA (Panula et al. 1998; Sondergaard et al. 2010). There is a lack of research comparing ACP and other canine CrCLR treatment modalities, their clinical outcomes, and impact on changes in the MMP-3 levels. This study compared the changes in the MMP-3 serum levels and the clinical outcomes between canine CrCLR cases treated by ACP injection or NSAIDs, by performing ELISA testing, orthopaedic examinations, and managing follow-up questionnaires before starting the initial treatment (d0), fourteen days after start of the treatment (d14), and twenty-eight days after the start of the treatment (d28). The results of the study support the hypothesis regarding the decline in the MMP-3 serum levels in dogs up to 28 days following treatment with both management procedures. Furthermore, the results demonstrate that the benefits to the dogs’ mobility and clinical outcomes are significantly higher when using a single injection of ACP versus NSAID management up to 14 days following the CrCLR with more sustained and higher benefits to the mobility and observed clinical outcomes. To the best of our knowledge, this is the first study to examine the effects of ACP injection vs oral NSAIDs on the serum MMP-3 levels and the clinical outcomes in the treatment of canine CrCLR.

The results of this study demonstrated significant changes in the serum MMP-3 levels in dogs with CrCLR treated with ACP injections and NSAIDs. As described in human medicine research, ACP has anti-inflammatory effects affecting the chondrocytes during OA (Unemori et al. 1991). Osteoarthritis negatively affects the articular matrix whose mechanism is associated with the MMP-3 action (Tchetina 2011; Kim 2019). The results of the significant differences between the MMP-3 serum levels at d14 and d28 in the cases of the CrCLR-treated canines with ACP indicate that ACP has a protective function on the articular matrix, with this effect lasting up to d28. On the contrary, the MMP-3 serum levels in dogs treated by oral NSAIDs were almost two times higher, indicating that ACP is more effective than NSAIDs in managing joint inflammation caused by CrCLR at d28. Additionally, the dogs that were treated with oral NSAIDs were administered a mixed supplement which can affect the cartilage and the joint environment. In the present study, it was detected that the serum MMP-3 levels of the canine CrCLR cases, treated by NSAIDs or ACP, decreased similarly in both groups up to d14; the main difference was revealed at d28 when the MMP-3 levels of the dogs treated by ACP decreased drastically. Kim et al. (2019) found that the mRNA expression levels of MMP-3 in dogs’ chondrocytes were lower after using ACP, but the effect was not measured over a longer period. To the author’s knowledge, this is the first research to measure the MMP-3 serum levels in canines with diagnosed CrCLR at three different time points in a span of twenty-eight days, across a range of different treatment modalities. The present study was not sufficiently powered to evaluate the correlation between the different degrees of OA and the selected treatment methods. This was due to the small number of patients in the groups and the limited range of OA degrees. These data may contribute to a more comprehensive understanding of the management of inflammatory processes in the stifle joint.

Painfulness and the associated lameness can be caused by OA (Vilar et al. 2018). In this study, the clinical outcomes were assessed by lameness scores, and the painfulness of manipulation scores at d0, d14, and d28. The evaluations were noted for all the dogs (*n* = 12) in the study. The data indicate that, in the cases of CrCLR treated with a single intra-articular injection of ACP, the lameness scores decreased up to 28 days, whereas, in the animals treated with oral NSAIDs, the lameness scores decreased up to 14 days, with a recurrence of lameness at d28.

It is important to note that the administration of NSAIDs for up to 14 days proved to be more advantageous in terms of the clinical outcomes. The reduction of this effect was observed only subsequent to this period when the NSAID usage was terminated. These results can be affected not only by the short-term activity of NSAIDs, but also by additional factors, such as the increased animals’ activity due to the improved clinical outcomes and less painfulness. In this case, the usage of NSAIDs may result in better clinical outcomes for up to 14 days, but this may also lead to a higher load to the limb and secondary damage to the joint. It should be noted that the same additional factors can also affect the results of the group treated by ACP.

The research indicated that no additional damage to the meniscus was sustained over the 28-day period, suggesting that the secondary damage to the stifle joint was unlikely. However, the short-term action of the NSAIDs may provide a valid explanation for the sudden worsening in clinical outcomes after 14 days in the group treated with NSAIDs.

Catarino et al. (2020) performed a gait analysis after four leukocyte-reduced ACP injections in canine CrCLR cases over three months and obtained results indicating improvement in the gait (Catarino et al. 2020). Our results of the painfulness of manipulation revealed a significant improvement in the patients treated with ACP. The patients treated with NSAIDs showed recurrent reactions to manipulations at d28, while those who were treated with ACP demonstrated minimal pain. Although our results revealed a significant reduction in the painfulness for dogs with CrCLR, the magnitude of this decline was greater in those treated with ACP than with NSAIDs at d28. Catarino et al. (2020) found out that ninety days after a single intra-articular ACP injection, the dogs were almost pain-free. Our results show that outcomes on lameness and painfulness of manipulations after the single intra-articular ACP injection are specified by longer-term lasting effects in comparison to the usage of NSAIDs.

There is a lack of significant data supporting the ACP effects to the overall patient’s general activity and quality of life. Follow-up questions, evaluating the patient’s mobility and quality of life, were collected from all the patient’s owners (*n* = 12) at d0, d14, and d28. To the best of our knowledge, this is the first study assessing the mentioned variables in dogs suffering from CrCLR. Raeissadat et al. (2021) researched the effects of an ACP injection on the quality of life in humans with stifle OA and obtained results showing that ACP increased the patients’ quality of life. Our results revealed significant changes in the follow-up scores in the cases of canine CrCLR treated with ACP or NSAIDs. The study results indicate that the treatment of canine CrCLR with ACP improved the quality of life and mobility in dogs compared to NSAIDs treatment after 28 days.

The present study is not without limitations, including the small sample size, lack of a control group, absence of tibial plateau angle measurements, low frequency of the evaluations, and overall short follow-up period. Furthermore, neither the veterinarian nor the owners were blinded to both treated groups.

Larger sample sizes, measurements of the tibia plateau angle, more frequent follow-up intervals, and longer overall follow-up period, and a randomised study design would enhance further studies and may facilitate a better comprehension of the effects of ACP as a treatment modality for canine CrCLR. Nonetheless, we believe that the findings of this study may significantly contribute to the further understanding of the management of canine orthopaedic diseases using novel treatment approaches.

The results of our research indicate that a single intra-articular ACP injection could be an effective and beneficial treatment method in terms of better clinical outcomes in cases of mild, moderate, and severe stifle OA caused by CrCLR, showing an extended duration of efficacy, and management of the inflammatory processes in the affected stifle joint, in contrast to oral NSAIDs.

It can be concluded that ACP, when administered once via injection into the stifle joint, has a more advantageous impact on the local inflammation of the stifle joint, orthopaedic parameters, and animal mobility. Furthermore, it has a more prolonged duration of action than oral NSAIDs in the treatment of canine CrCLR, lasting up to twenty-eight days.
